# Nickel Allergy Is a Risk Factor for Endometriosis: An 11-Year Population-Based Nested Case-Control Study

**DOI:** 10.1371/journal.pone.0139388

**Published:** 2015-10-06

**Authors:** Jin-Sung Yuk, Jong Seung Shin, Ji-Yeon Shin, Eunsuk Oh, Hyunmee Kim, Won I. Park

**Affiliations:** 1 Department of Obstetrics and Gynecology, School of Medicine, Eulji University, Daejeon, South Korea; 2 Department of Obstetrics and Gynecology, MizMedi Hospital, Seoul, South Korea; 3 Department of Obstetrics and Gynecology, Hyundai UVIS hospital, Incheon, South Korea; 4 Department of Preventive Medicine, School of Medicine, Eulji University, Daejeon, South Korea; 5 Department of Internal Medicine, MizMedi Hospital, Seoul, South Korea; 6 Department of Family Medicine, MizMedi Hospital, Seoul, South Korea; University of Malaya, MALAYSIA

## Abstract

**Background:**

A cross-sectional study has reported that nickel allergy is associated with endometriosis. However, causal studies of this association are limited.

**Objective:**

The objective of this study was to compare the prevalence of nickel allergy in women with and without endometriosis.

**Methods:**

We used a National Health Insurance Service (NHIS) sample cohort dataset that included approximately 1 million individuals from South Korea; the data were obtained between January 01, 2002, and December 31, 2013. We selected the endometriosis group according to diagnosis code (N80.X), surgery codes, and drug codes during the years 2009~2013. The controls were randomly matched to the endometriosis patients at a ratio of 4:1 by age and socioeconomic status. Patients with nickel allergy were defined in the cohort dataset as those with a simultaneous diagnosis code (L23.0) and patch test code during 2002~2008.

**Results:**

In total, 4,985 women were selected from the NHIS cohort database and divided into an endometriosis group (997 women) and a control group (3,988 women). The number of patients with nickel allergy in the endometriosis group was eight (0.8%), and that in the control group was thirteen (0.3%). After adjustment for age and socioeconomic status, the rate of nickel allergy in was higher in the endometriosis group than in the control group [odds ratio: 2.474; 95% confidence interval: 1.023~5.988; p = 0.044].

**Conclusions:**

We found that nickel allergy is a risk factor for endometriosis.

## Introduction

Endometriosis is an estrogen-dependent disease that causes pelvic pain and subfertility in 6–10% of women [1]. Although debate continues regarding the cause of endometriosis, the central theory involves the retrograde flow of endometrial cells into the pelvic cavity during menstruation [1,2]. However, the main weakness of this theory is that only 6–10% of all women have retrograde menstruation [1]; thus, some complementary theories are needed. One hypothesis is that environmental substances such as dioxin, polychlorinated biphenyls and organochlorine pesticides may cause endometriosis [3,4]. Another hypothesis is that changes in the immune response might affect the survival of endometrial cells external to the endometrium [5,6].

Recently, using national claims data in South Korea, Yuk et al. demonstrated a high rate of nickel allergy in women with endometriosis [7]. Because nickel allergy involves features of environmental exposure and the immune response, there may be a relationship between nickel allergy and the pathogenesis of endometriosis [8]. However, the study of Yuk et al. was not a causal study but rather a correlational study. Thus, it is unclear which disease precedes the other.

The aim of this nested case-control study was to evaluate the prevalence of nickel allergy in women with and without endometriosis using national claims cohort data collected from 2002 to 2013. To the best of our knowledge, this is the first nested case-control study to assess the causal relationship between nickel allergy and endometriosis.

## Materials and Methods

### Sample

We used a sample cohort dataset provided by the National Health Insurance Service (NHIS) [9]. These data corresponded to approximately 1 million individuals selected randomly from almost all South Koreans, totaling 45 million people, with national claims data for the period from January 1, 2002, to December 31, 2013. The included variables were gender, 5-year age group, socioeconomic status (with subjects divided into 10 categories based on income), diagnosis code, surgery code, drug prescription data (drug name, dosage, and date of prescription) and billing code.

### Selection of subjects

**Fig 1:**
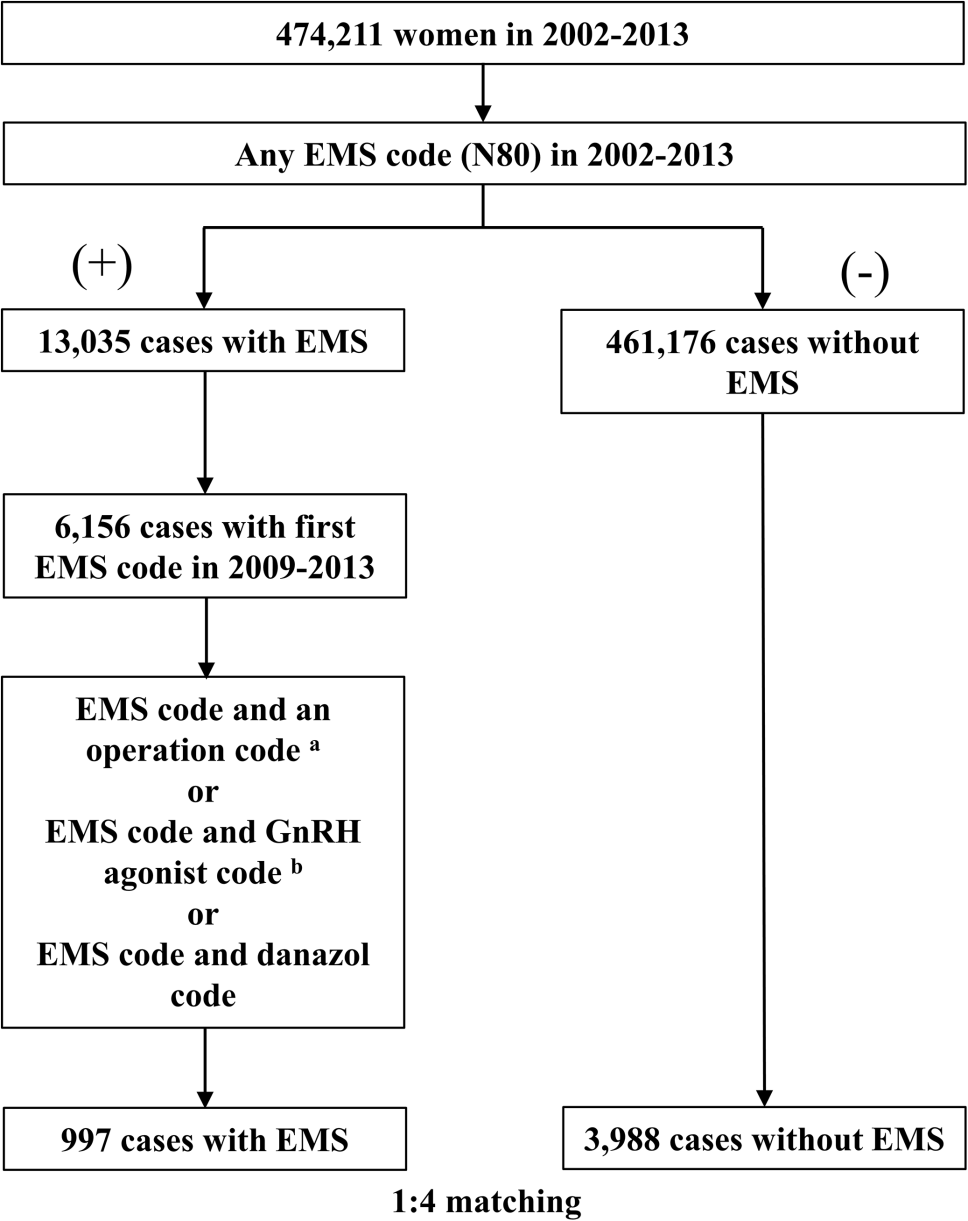
Flow chart representing the selection procedure based on the NHIS cohort data set from 2002–2013. ^a^ Surgery codes: R4122, R4160, R4165, R4166, R4170, R4181, R4182, R4183, R4331, R4332, R4341, R4342, R4345, R4421, R4430, and R4435. ^b^ Gonadotropin-releasing hormone agonist codes and danazol codes: 182602BIJ, 182604BIJ, 244902BIJ, 167202BIJ, 167201BIJ, 198501CSI, 140301ACH, and 140302ACH. Abbreviations: EMS, Endometriosis; NHIS, National Health Insurance Service; GnRH, Gonadotropin-releasing hormone.

We used the International Classification of Diseases (ICD) 10^th^ edition to extract the endometriosis group and the control group. We selected the endometriosis group as follows. We excluded patients with any endometriosis diagnosis code (N80.X) prior to 2009 from the endometriosis group. We selected patients with an endometriosis diagnosis code (N80.X) assigned between 2009 and 2013 from the NHIS sample cohort data collected during 2002–2013 (Fig 1). Among these endometriosis patients, we selected patients who simultaneously had an endometriosis diagnosis code (N80.X) and one or more of the following surgery codes [R4122 (myomectomy), R4160 (pelvic adhesiolysis), R4165 (fulguration), R4166 (foreign body removal), R4170 (metroplasty of uterine anomaly), R4181 (Kustner operation), R4182 (manual reduction), R4183 (total hysterectomy), R4331 (unilateral adnexectomy), R4332 (bilateral adnexectomy), R4341 (ligation of fallopian tubes), R4342 (surgical fulguration of oviducts), R4345 (laparotomy), R4421 (extirpation of benign adnexal tumor), R4430 (ovarian wedge resection), R4435 (incision and drainage of ovarian cyst)]; patients with a simultaneous endometriosis diagnosis code (N80.X) and gonadotropin-releasing hormone (GnRH) agonist code [182602BIJ (leuprolide acetate), 182604BIJ (leuprolide acetate), 244902BIJ (triptorelin acetate), 167202BIJ (goserelin acetate), 167201BIJ (goserelin acetate), 198501CSI (nafarelin)]; and patients with a simultaneous endometriosis diagnosis code (N80.X) and danazol code (140301ACH, 140302ACH) to increase diagnostic accuracy. Among patients without any endometriosis diagnosis code (N80.X) during 2002~2013, the controls were randomly matched to the endometriosis patients at a ratio of 4:1 by 5-year age group and socioeconomic status (Fig 1). Patients with nickel allergy were identified as those who simultaneously had a nickel allergy diagnosis code (L23.0) and a test code [patch test (E7130), skin prick test (E7151, EY853), intradermal test (E7152, EY854)] among the cohort dataset during 2002–2008.

### Statistics

Data management and analysis were performed using the R statistical software (version 3.0.3; Vienna, Austria). Significance was considered for p-values under 0.05. All of the statistical analyses were performed using two-tailed tests. The chi-squared test or Fisher’s exact test was applied to compare categorical differences between two groups. After adjustments for age, socioeconomic status and sampling year, conditional logistic multivariate analysis was performed to determine the effects of nickel allergy on endometriosis.

### Ethics statement

For information protection, all of the patients received an anonymous identification code in the sample data provided by the NHIS. The authors could not identify any patients in the sample data. Thus, we did not require the approval of the Institutional Review Board, in accordance with the guidelines of the Institutional Review Board of MizMedi Hospital.

## Results

A total of 4,985 women were selected from the NHIS cohort database, which included approximately 1 million individuals from 2002 to 2013. Among the 4,985 women, the endometriosis group included 997 women. The control group included 3,988 women who were matched to the endometriosis patients according to 5-year age group and socioeconomic status at a ratio of 4:1 (Fig 1 and Table 1). The mean age in both groups was 42.6±9.3 years in 2013. The number of patients with nickel allergy in the endometriosis group was eight (0.8%), and the number of patients with nickel allergy in the control group was thirteen (0.3%). After adjusting for 5-year age group and socioeconomic status, the prevalence of nickel allergy was higher in the endometriosis group than in the control group [odds ratio (OR): 2.474; 95% confidence interval (CI): 1.023–5.988; p = 0.044] (Table 2).

**Table 1:** Characteristics of patients in the endometriosis group and the control group, 2002–2013.

	EMS group	Control group	Total	p-value
Number of patients	997	3,988	4,985	
2009 ^a^	183			
2010 ^a^	198			
2011 ^a^	198			
2012 ^a^	201			
2013 ^a^	217			
Mean age group ^b^	8.5±1.8	8.5±1.9	8.5±1.8	1
Mean age (years) ^c^	42.6±9.2	42.6±9.3	42.6±9.3	
Mean group socioeconomic status ^d^	6.0±2.9	6.0±2.9	6.0±2.9	1
Nickel allergy				0.052 ^e^
Present	8 (0.8%)	13 (0.3%)	21	
Not present	989 (99.2%)	3975 (99.7%)	4964	

^a^ First diagnosis of EMS was made in the year noted.

^b^ Mean age groups per 5 years in 2013. 1; 0–4 years, 2; 5–9 years,……., 17; 80–84 years, 18; 85 years+.

^c^ The mean age was calculated from the mean age groups per 5 years.

^d^ Mean socioeconomic status groups in 2013. 1; upper 0–9%, 2; upper 10–14%,……., 9; upper 90–94%, 10; upper 95%+.

^e^ Fisher’s exact test

Data are presented as numbers or means ± standard deviations

**Table 2:** Multivariate logistic regression analysis of endometriosis and nickel allergy.

Adjustment	Crude OR		Adjusted OR ^a^	
	OR (95% CI)	p-value	OR (95% CI)	p-value
Age group per 5 years	1.001 (0.963~1.038)	1	1.001 (0.964~1.039)	0.977
Socioeconomic status group (decile)	1.000 (0.976~1.024)	1	1.000 (0.976~1.024)	0.977
Nickel allergy	2.473 (1.022~5.984)	0.045	2.474 (1.023~5.988)	0.044

^a^ Covariates: Age group per 5 years, socioeconomic status group (decile), nickel allergy.

CI, Confidence interval; OR, Odds ratio

## Discussion

Nickel is a silvery-white metal that is mainly used as an alloy with iron, copper, chromium, and zinc, and it is used for plating, coins, batteries, jewelry, buttons, eyeglasses, zippers, valves, heat exchangers, and promoters [10]. Nickel allergy has a prevalence that ranges from 10–23% among women [11,12], and it is one of the most common forms of allergic contact dermatitis, which results in itching, redness, rash, dryness, swelling and blisters [13].

Our case-control study suggests that nickel allergy is a risk factor for endometriosis. To date, there has been little quantitative analysis of the relationship between nickel allergy and endometriosis. Recently, one study showed that nickel concentrations in the blood of patients with endometriosis were higher than those in a control group [14]. More recently, Yuk et al. reported a positive relationship between nickel allergy and endometriosis using a cross-sectional population-based study [7]. However, no previous study has evaluated the causal link between nickel allergy and endometriosis.

It is not yet clear why nickel allergy is a risk factor for endometriosis, although the studies discussed below could provide clues on this topic. The occurrence of nickel allergy increases with exposure to nickel. As an example, one study has reported that women, who are frequently exposed to nickel accessories, show a higher prevalence of nickel allergy than men, who are not [8]. In addition, there is a higher prevalence of nickel allergy among hairdressers, who frequently use nickel alloy scissors, and retail clerks, who handle coins more frequently than the general population [8]. Because frequent exposure to nickel occurs throughout daily life, nickel allergy has been associated with high nickel levels in the blood [15,16]. Thus, a high blood concentration of nickel in patients with nickel allergy might cause endometriosis.

If this relationship is true, what characteristics of nickel might cause endometriosis? Although we do not know the exact reason, the estrogenic characteristics of nickel might affect endometriosis [17,18]. Specifically, nickel can bind the metal-binding sites on the estrogen receptor (ER) in vitro [19], and nickel has also been shown to stimulate ERα expression and activity in breast cancer cells [20]. Furthermore, nickel concentrations in blood, urine, hair, and breast tumor tissues have been positively correlated with breast tumors [18]. Several such lines of evidence suggest that nickel has estrogenic characteristics. Similarly, certain chemicals have estrogen-like characteristics [21,22], and estrogens have an important effect on endometriosis [1]. In conclusion, nickel allergy is associated with high nickel blood levels, and these high nickel levels might affect the development of endometriosis due to the estrogenic effects of nickel.

As discussed earlier, endometriosis and nickel allergy may share a common pathogenesis [8]. The underlying mechanism of nickel allergy is delayed cell-mediated hypersensitivity [23]. Similarly, endometriosis is related to an increased T-helper 1 response, which plays a key role in cell-mediated hypersensitivity [24].In addition, certain autoimmune diseases, such as autoimmune thyroiditis, involve increased lymphocyte reactivity to nickel [25].Nickel allergy predominantly affects women [11]. These findings indicate that nickel may exhibit autoimmune characteristics. Given the autoimmune characteristics of endometriosis, endometriosis and nickel allergy may share common features [26]. However, the some characteristics of the immune response in each disease are different; nickel allergy is an immunologic cell-mediated response involving a hapten (a low-molecular-weight compound) [27], whereas endometriosis has not been associated with any trigger chemicals, such as haptens, to date. Additionally, endometriosis is not related to other allergic diseases such as allergic rhinitis, atopic dermatitis or even other forms of contact dermatitis, with the exception of nickel allergy, according to a cross-sectional population-based study [7]. It thus remains unclear whether immune dysregulation caused by nickel exposure is a causal mechanism in the development of endometriosis.

Our results should be interpreted with caution. Because the prevalence of women in whom nickel allergy preceded endometriosis was only 0.8%, the influence of nickel allergy in the pathogenesis of endometriosis may be of low clinical importance. However, most cases of nickel allergy are diagnosed clinically based on history and physical examination findings [28]. In contrast, in our study, we restricted our cohort of patients with nickel allergy to women who had undergone patch testing to increase the accuracy of the diagnosis. Therefore, the number of patients with nickel allergy might be underestimated in our study. In support of this possibility, the prevalence of nickel allergy was only 0.3% in the control group. Considering that the prevalence of nickel allergy ranges from 10–23% in the general population, the true prevalence of nickel allergy in our study might be much higher than 0.3% [11,12]. Therefore, although it is impossible to assert that nickel allergy affects all patients with endometriosis, nickel allergy might be one of several causes influencing the development of endometriosis.

This study also had several limitations. First, there was a lack of patient data regarding the histological findings and staging of endometriosis. To complement this point, only endometriosis patients who had undergone surgery or received a GnRH agonist or a danazol injection were included in our study. Despite these efforts, there may be some limitation to the analysis of endometriosis, especially regarding stage. Second, there is a possibility that patients were miscoded with respect to their diagnosis, examination, surgery or administered drugs. However, because the possibility of miscoding was similar in the endometriosis group and the control group, the influence of this possibility may be limited. Third, our data did not include the stage of endometriosis, obstetric history or occupation. Thus, we could not adjust for those variables in our logistic regression analysis.

In conclusion, we found that nickel allergy is a risk factor for endometriosis. Further study with greater focus on the irritant features or immune response to nickel is suggested.
